# Large-Scale Analysis of Drug Side Effects via Complex Regulatory Modules Composed of microRNAs, Transcription Factors and Gene Sets

**DOI:** 10.1038/s41598-017-06083-5

**Published:** 2017-07-20

**Authors:** Xiaodong Jia, Qing Jin, Xiangqiong Liu, Xiusen Bian, Yunfeng Wang, Lei Liu, Hongzhe Ma, Fujian Tan, Mingliang Gu, Xiujie Chen

**Affiliations:** 1 College of Bioinformatics Science and Technology, Harbin Medical University, Harbin, China; 2 Joint Laboratory for Translational Medicine Research, Beijing Institute of Genomics, Chinese Academy of Sciences & Liaocheng People’s Hospital, Liaocheng, China; 3 CAS Key Laboratory of Genome Sciences and Information, Beijing Institute of Genomics, Chinese Academy of Sciences(CAS), Beijing, China

## Abstract

Identifying the occurrence mechanism of drug-induced side effects (SEs) is critical for design of drug target and new drug development. The expression of genes in biological processes is regulated by transcription factors(TFs) and/or microRNAs. Most of previous studies were focused on a single level of gene or gene sets, while studies about regulatory relationships of TFs, miRNAs and biological processes are very rare. Discovering the complex regulating relations among TFs, gene sets and miRNAs will be helpful for researchers to get a more comprehensive understanding about the mechanism of side reaction. In this study, a framework was proposed to construct the relationship network of gene sets, miRNAs and TFs involved in side effects. Through the construction of this network, the potential complex regulatory relationship in the occurrence process of the side effects was reproduced. The SE-gene set network was employed to characterize the significant regulatory SE-gene set interaction and molecular basis of accompanied side effects. A total of 117 side effects complex modules including four types of regulating patterns were obtained from the SE-gene sets-miRNA/TF complex regulatory network. In addition, two cases were used to validate the complex regulatory modules which could more comprehensively interpret occurrence mechanism of side effects.

## Introduction

Drug side effects (SEs), which is the main reason of drug development failures, are usually regarded as an undesirable secondary effect and serious adverse influence, which occurs in addition to the desired therapeutic effect of a drug or medication. Understanding the occurrence mechanism of side effects is vital for prevention of side effects and rational design of drug. It has been known that drug side effects are regulated by gene, miRNA and TF. Having a good understanding about the complex regulating relations composed of these factors will benefit researchers to interpret the occurrence mechanism of side effects more comprehensively.

Previously, research about the side effects mechanism is mainly focused on drug off-target proteins. The interaction between drugs and off-targets can change the states of biological processes, and further induce the occurrence of side effects. Through combining the GeneChip expression data about the toxic doses of the drug, many researchers performed the functional enrichment analysis, in order to interpret the occurrence mechanism of drug side effects. Sejoon Lee mapped the differentially expressed genes into the biological processes, for revealing the side effect mechanism^1^. Keiser *et al*. utilized chemical similarity to acquire off-targets for a known drug^2^. Wang *et al*. studied the relationship between the incidence of side effect and the network distance of drug target and disease-related genes^3^, and further identified the risk proteins. Furthermore, Chen *et al*. stated the network topology characteristics of Adverse Drug Reaction (ADR)-related protein and expounded the concomitant phenomenon of ADRs since they share the common ADR-related proteins^4^. By reviewing the previous studies, it was found that most researchers illustrated the generating processes of drug side effects only from the view of functional genes. It has been widely suggested that the functional genes implicated in biological processes are regulated by its target miRNAs and TFs. The composite regulatory modules are composed of TFs, miRNA and biological processes. miRNAs, TFs and their target mRNAs (genes) can interact with each other, and form a complex regulated system to the biological processes inducing side effects. Thus studying the molecular mechanism of side effects only from the aspect of genes/gene sets is far from enough, and discovering the mechanism from the interaction among TFs, miRNAs and biological processes will be more comprehensive. To our knowledge, this is the first article to study the relationship among miRNAs, TFs and biological processes in side effects.

**Figure 1:**
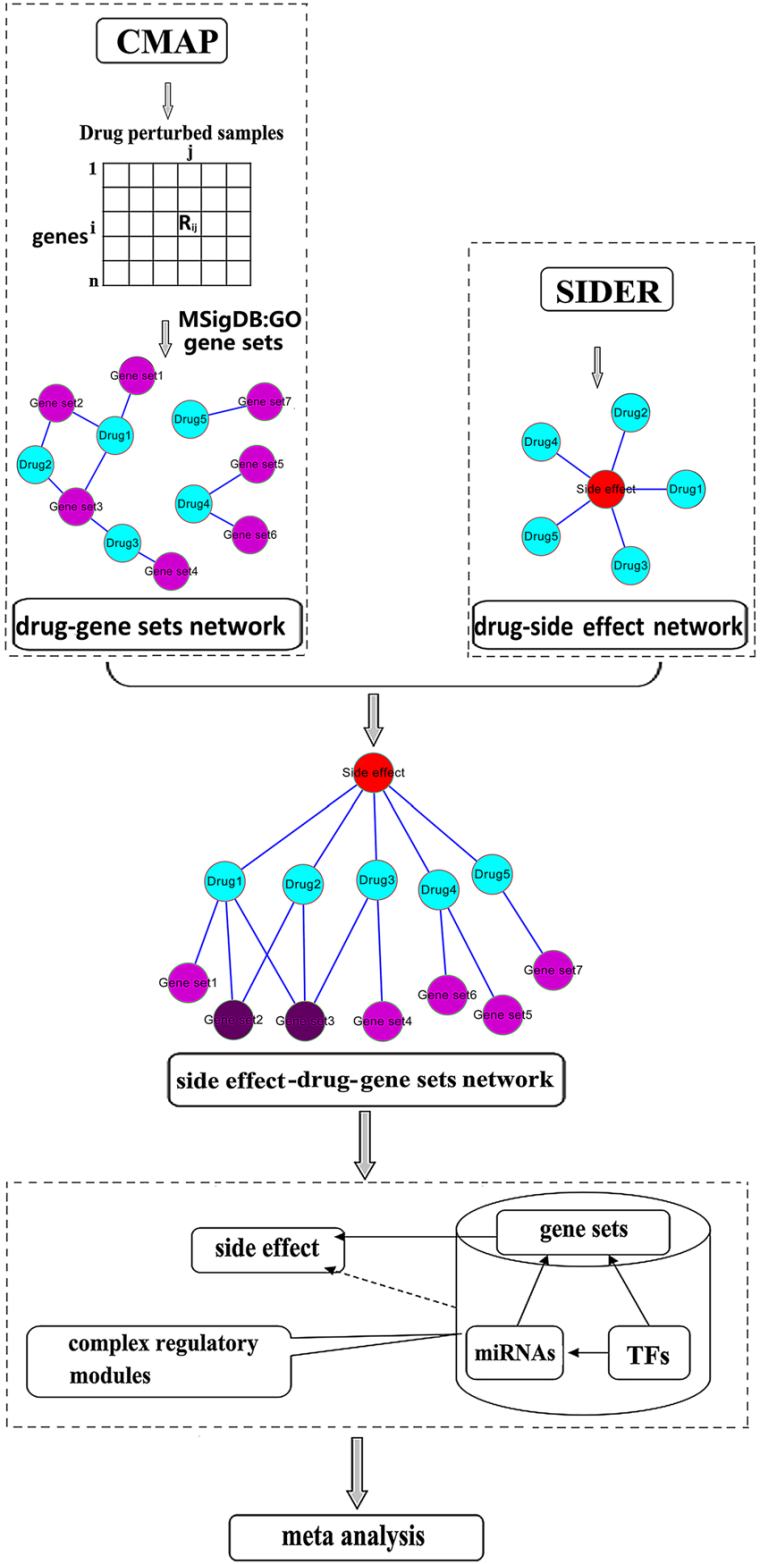
The specific work flow. The red nodes represent side effects, the cyan nodes represent drug, the purple nodes represent gene sets, the deep purple nodes represent side effect-related gene sets. Firstly, we enriched the drug related genes to GO gene sets to establish drug-gene sets network. Secondly, in term of the drug-side effect network and drug-gene sets network, we built a side effect -drug-gene sets network and identified the significant side effect-gene set pairs. Thirdly, we set up a side effect-gene sets-miRNA(TF)composite network after enriching the gene sets to the miRNA(TF). Finally, we extracted composite regulatory modules including four regulated patterns of all side effects from the directed regulated network composed of gene set, miRNA and TF. And took Pneumonia and Neutropenia as example for comprehensively interpreting occurrence mechanism of side effects.

In this study, a directed complex regulatory network composed of TFs, miRNAs and biological processes was constructed, and the relationships among many SE-related complex regulatory patterns including gene sets, miRNAs and TFs were also discovered. The specific work flow is shown in Fig. 1. This study used multiple bioinformatics methods to interpret the mechanism of side effects from a new perspective, and contributed the guidelines to the drug development and clinical application.

## Results

Firstly, utilizing the Fisher’s enrichment analysis, drug-gene set network (Supplementary Data 1) was acquired, and SE-drug-gene set complex relationship was established through connecting the drug-gene sets network with SE-drug network, and then the SE-related gene set network (Supplementary Fig. 1, Supplementary Data 2) was obtained through randomly perturbing drugs. The regulatory relationship was analyzed based on the frequency distribution of the regulatory number, and the strong correlation modules of gene sets and side effects. Afterwards, the gene sets of SE-gene set relationships were enriched into miRNAs and TFs, and then SE-gene set-miRNA (TF) complex network was constructed. The complex regulatory modules for each side effect were extracted from the complex network. Four regulatory patterns were identified, and the possible occurrence mechanism of side effects regulated by multiple patterns of complex regulatory modules was analyzed from an overall perspective for every side effect.

### SE–gene set relationships

The differentially expressed genes of 72 drugs were enriched into GO gene sets, and 3830 significant drug-gene sets pairs including 72 drugs and 943 GO gene sets were obtained. To construct the SE-drug-gene set complex network, the drug-gene set relationships and drug-SE relationships were integrated based on drugs. Besides, in order to investigate how many drugs with the same processes in the complex network were significant, SE-drug relationships were randomly generated. Since different gene sets generated diverse drug-gene set relationships, thus the threshold of gene set-related drugs were different. Table 1 showed the total number of drugs causing a side effect and how many co-occurring drugs were significant in the total number of drugs in GO gene sets. When 8 kinds of drugs causing side effect were applied, common significant GO gene sets in more than 4 drugs are significant to side effect.

**Table 1:** SE-related gene sets threshold.

Number of total drugs causing a side effect	Co-occurrences
2,3,4	2
5,6,7,	3
8,9,10,11,12	4
13,14,16	5
19,21	6
23,25	7

**Figure 2:**
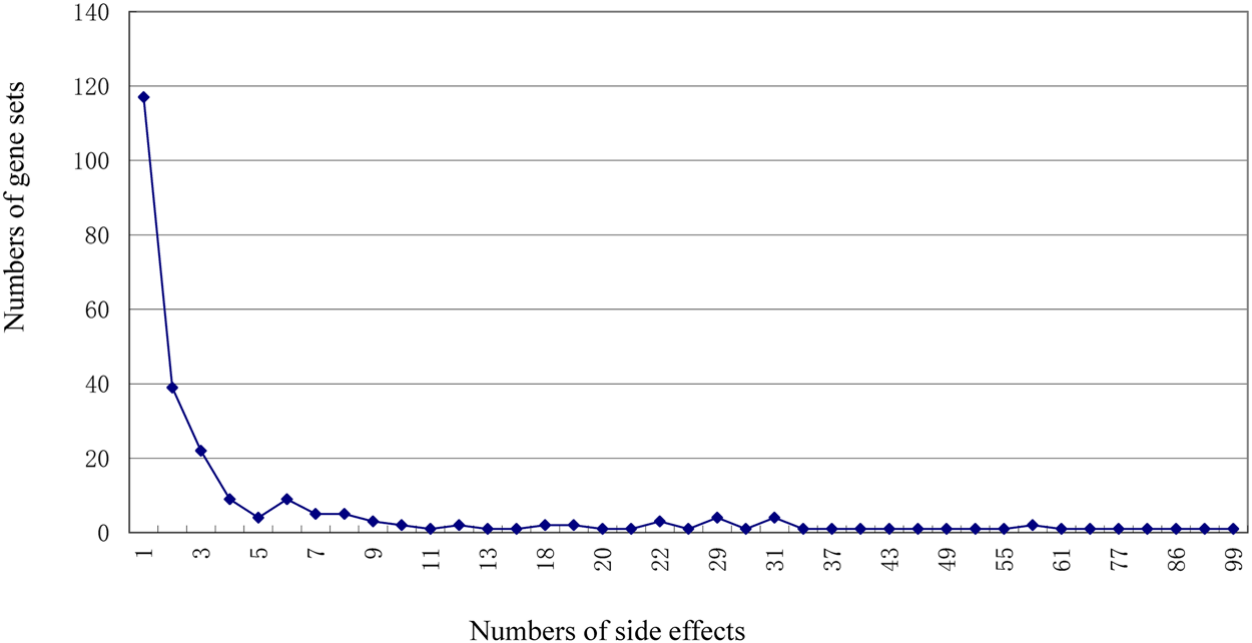
The frequency distribution of gene sets controlling the side effects. The horizontal axis indicated the number of side effects, and the vertical axis indicated the number of gene sets inducing corresponding side effects. The results showed that each gene set regulated 8.06 SEs averagely, and most of gene sets were associated with the minority of side effects.

Based on the threshold of gene set-related drugs, eventually, 2064 significant SE-gene set relationships were obtained, including 117 SEs and 256 gene sets. To characterize the relationships between gene sets and side effects, the frequency distribution of gene sets controlling side effects were analyzed (Fig. 2). In Fig. 2, the horizontal axis indicated the number of side effects, and the vertical axis indicated the number of gene sets regulating correspondent side effects. The results showed that each gene set regulated 8.06 SEs averagely, and most of gene sets were associated with minority of side effects. 178 (69.5%) gene sets regulated three SEs at most, and among them, 117 (45.7%) gene sets regulated only a single SE which was defined as the special gene sets of the side effects. The special gene sets mainly controlled four kinds of SEs including lightheadedness, pneumonia, thrombocytopenia and vaginal discharge. Besides, a small number of gene sets controlled multiple side effects (more than three SEs), and the gene sets related with more than 50 SEs were associated with the function of cell membrane such as G protein coupled receptor signaling pathway and integral to plasma membrane etc. (Supplementary Data 3).

**Figure 3:**
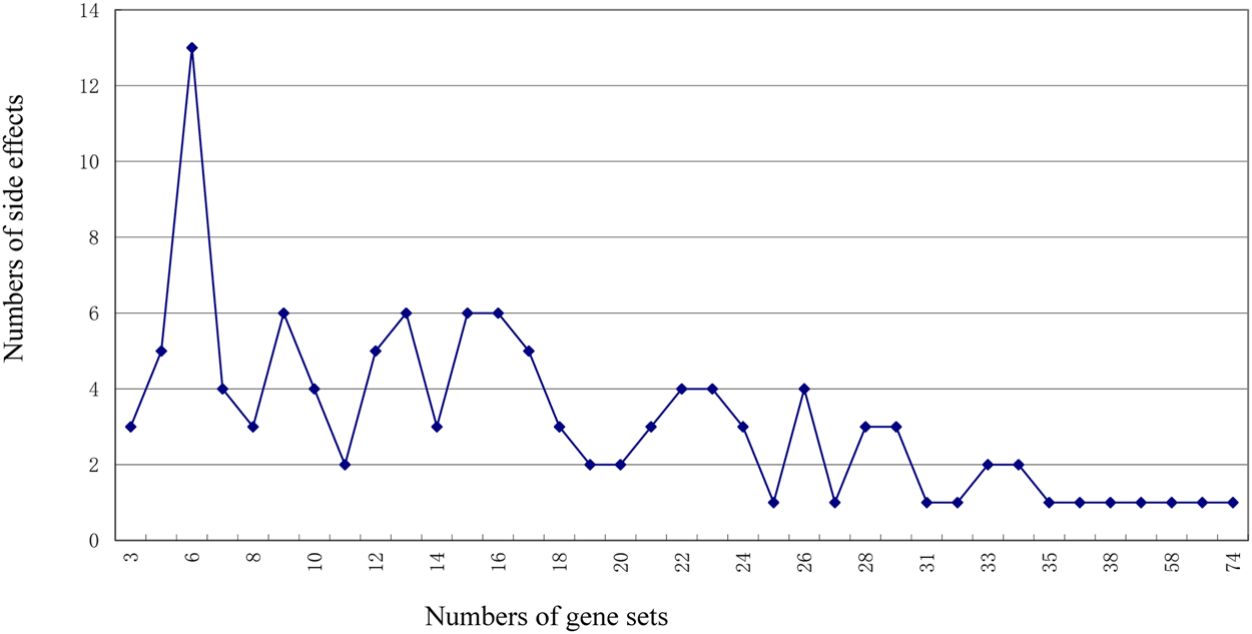
The frequency distribution of side effect regulated by gene sets. The horizontal axis means the number of gene sets which regulated a side effect, and the vertical axis means the number of side effects. The results showed that each side effect was controlled by 17.64 gene sets averagely. Most side effects were related with numerous gene sets, but only three SEs were controlled by three gene sets, and other side effects were associated with at least five gene sets (the largest up to 74).

It is known that the occurrence of any side effect is a complex process of body reaction. To inquiry how many gene sets controlled the occurrence of a side effect, this study made a further investigation about the frequency distribution of side effects regulated by gene sets (Fig. 3). In Fig. 3, the horizontal axis means the number of gene sets which regulated a side effect, and the vertical axis means the number of side effect. The results showed that each side effect was controlled by 17.64 gene sets averagely. Most side effects were related with numerous gene sets, but only three SEs were controlled by three gene sets, and other side effects were associated with at least five gene sets (the largest up to 74). The fact that side effects were related to multiple gene sets indicated that the occurring mechanism of side effects was actually very complex. And the side effects tended to occur more easily, because side effects would be caused as long as any of the gene sets were affected by drugs. It was also found that four SEs associated with the largest number of gene sets were also the side effects that regulated 117 special gene sets. For example, the occurrence of vaginal discharge was related with 74 gene sets including 37 special gene sets; thrombocytopenia was related with 70 gene sets including 15 special gene sets; pneumonia was associated with 58 gene sets including 26 special gene sets; lightheadedness was involved in 51 gene sets including 10 special ones. Meanwhile, non-special gene sets were related with the occurrence of multiple side effects, which meant multiple different side effects shared these gene sets. Because of this, these gene sets may induce the simultaneous occurrence of side effects sharing these gene sets (Supplementary Data 4).

**Figure 4:**
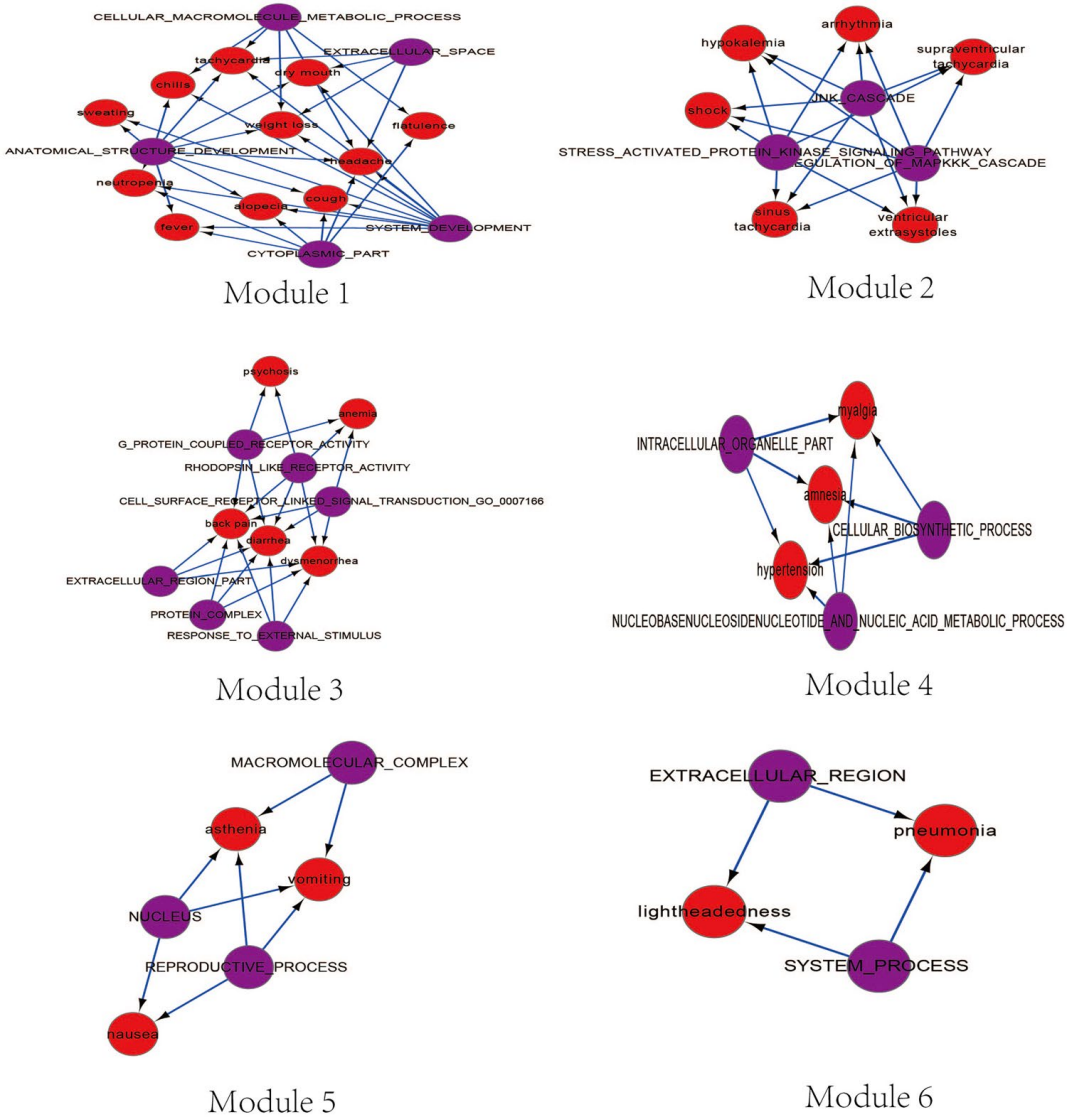
Side effects-gene sets modules. Purple points represent gene sets and red points represent side effects. The side effects within each SE-gene set module often occur simultaneously because they share the same gene sets, e.i. with similar SE occurrence mechanism.

To further identify the close relationship between side effects and gene sets, and the potential accompanying relationship among side effects. Utilizing the plugin MCODE of Cytoscape, six highly close SE-gene set modules were obtained (Fig. 4). The genes sets in the same module played similar roles and were shared by the side effects within the module. Taken module 2 as an example, the gene sets in module 2 were the functional gene sets relevant with protein kinases and signaling pathways, and the side effects within module 2 were heart related illnesses such as arrhythmia, supraventricular tachycardia (SVT), sinus tachycardia, ventricular extrasystoles, shock and hypokalemia. Three gene sets in module 2 were associated with these above six side effects. Activating protein kinase C can promote the ventricular action potential duration (APD) and thus cause the arrhythmia^5^, Meanwhile, creatine kinase (CK) has been suggested to be bound up to myocardial damage induced by SVT^6^. These results indicated that the side effects sharing the gene sets can simultaneously occur, once the states of gene sets were changed by some signals. Therefore, strict attentions should be paid on the chemical substances of drugs that influenced the gene sets.

### SE-gene set-miRNA/TF network

**Figure 5:**
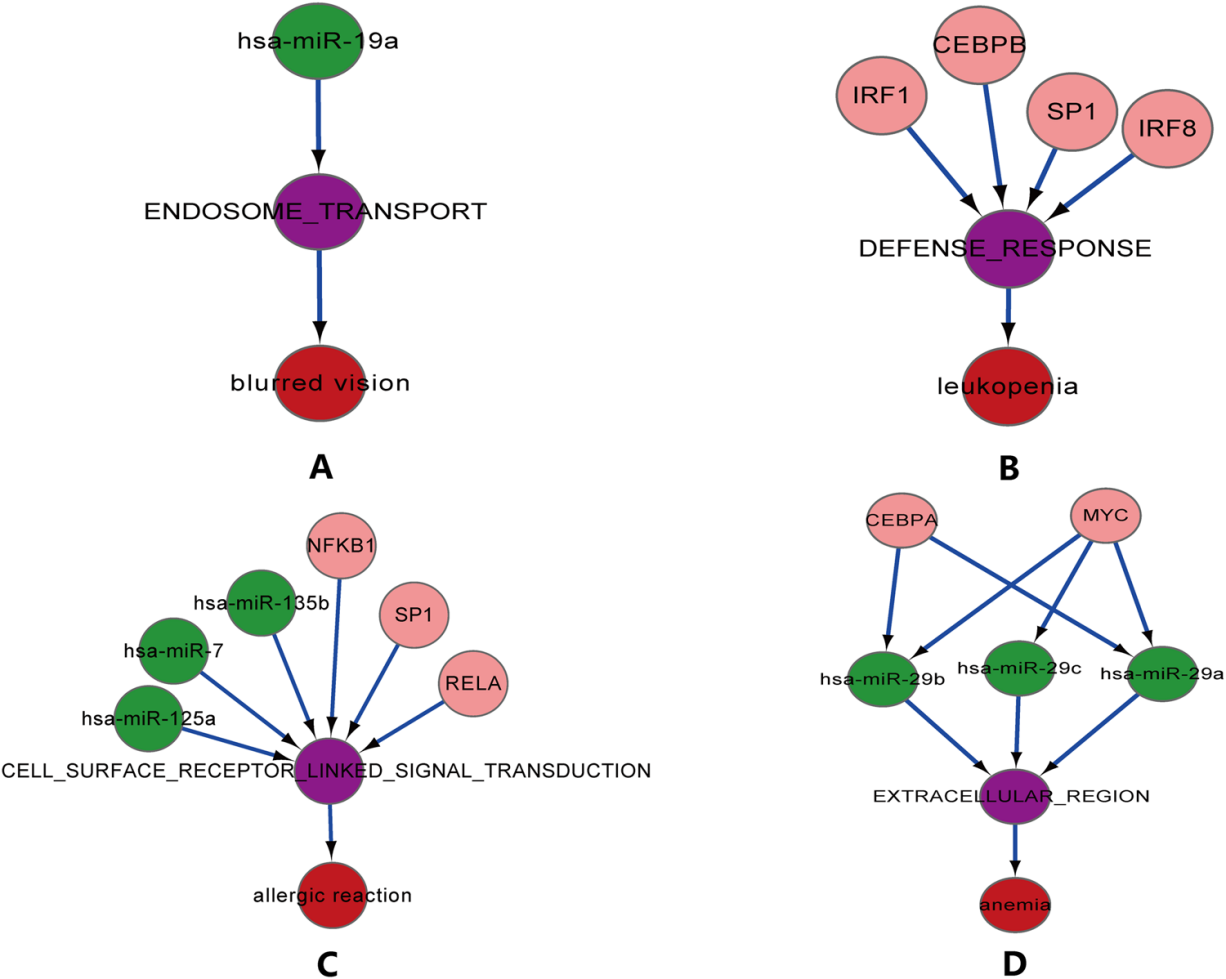
Examples of four regulatory patterns. The red nodes represent side effects; the purple nodes represent gene sets; the green nodes represent miRNAs and the pink nodes represent TFs. (**A**) The first regulatory pattern; (**B**) The second regulatory pattern; (**C**) The third regulatory pattern; (**D**) The last regulatory pattern.

We enriched gene sets into miRNA (or TF), a total of 1671 SE-gene set pairs, 1072 gene set-miRNA pairs and 2480 gene set-TF pairs were obtained. Then the SE-gene set-miRNA (TF) complex network (Supplementary Fig. 2, Supplementary Data 5) were established, including 117 SEs, 142 gene sets, 198 miRNAs, 404 TFs and 466 TF-miRNA regulatory pairs. For each side effect, SE -special complex regulatory module was extracted, which was composed of four relationships (Fig. 5): SE-gene set-miRNA relationships, SE-gene set-TF relationships,SE-gene set-miRNA (and TF) and SE-gene set-miRNA-TF relationships. Complex regulatory module sets of 117 side effects were acquired from the SE-gene set-miRNA (TF) network (Supplementary Data 6).

The first regulatory pattern indicated that gene sets inducing SEs were only controlled by post-transcriptional negative regulation of miRNA. In this pattern, there were 19 SE-gene set relationships containing 13 SEs and 10 gene sets. These gene sets were regulated by 13 miRNAs which meant each gene set was controlled by 3.05 miRNAs. The first pattern utilized the live link-up between miRNAs and functional gene sets to explore the occurrence mechanism of side effects. For example, blurred vision is the loss of visual acuity resulting in a loss of ability to see small details and also a usual side effect owning to relax to the ciliary muscle of the eye after taking some medications. It is known that ciliary muscle has sympathetic and parasympathetic innervation, this means that the sympathetic and the parasympathetic pathways are involved in the regulation of relaxation and contraction of the muscle. When the eye needs to fine focus, the contraction of the ciliary muscle causes compression of the eye lens to accommodate this. Typically, closer or smaller objects require more effort to see which is achieved by contraction of the ciliary muscle compressing the lens. While taking too much amphetamine like substances lead to excessive activation of the sympathetic pathway and the excess noradrenaline (norepinephrine) released leads to the constant activation of the beta-2 sympathetic receptors of the ciliary muscle and as the sympathetic pathway is the dominant one in that muscle, the muscarinic pathway is left unable to focus the eye by contracting the ciliary muscle^7^. Based on our method, we found that blurred vision was highly related with the gene sets of endosome transport, which coincides with the norepinephrine releasing, transporting and binding to the beta-2 sympathetic receptors of the ciliary muscle, these biological processes are mediated by endosome signal transduction^8^. Meanwhile we also found that the gene sets of endosome transport were negatively regulated by hsa-miR-19a in the posttranscriptional stage, which the miRNA altered the states of gene sets of endosome transport through their target proteins such as ABCA1, ABCA1 abnormality leads to two vision phenotype abnormal retina morphology and abnormal retinal pigmentation^9^. Therefore, integrating miRNAs and their common regulatory gene sets enable us to identify the occurrence mechanism of drug-induced side effects.

The second pattern is that side effects were controlled by gene sets, gene sets were only regulated by TF before transcription. In this pattern, there were 1256 side effect-gene sets relationships containing 117 side effects and 80 gene sets which were regulated by 163 TFs, i.e. one gene set was controlled by 6.55 TFs on average. And some transcription factors affect the transcription of many genes and induce more side effects occurrence. For example, SP1 transcription factor was involved in 783 side effect-gene sets relationships which covered 107 SEs and 26 gene sets. From Fig. 5B, we found that the occurrence of leukopenia was related to defense response gene sets which were regulated by CEBPB, IRF protein family, and the transcription factor SP1, etc. It has been generally acknowledged that the defense response of body can be activated when the body is affected by foreign materials or appears any damages. The influenced cellular quantity and function in the antiviral therapy and host defense responses, however, give rise to the abnormal immune response manifesting leukocytopenia^10^. CEBPB could exert important effect in inflammatory and immunological responses, especially in the Interleukin 6 (il-6)-mediated gene expression^11^, and also SP1 can be involved in variety processes such as immune response^12^. The IRF (interferon regulatory factor) family, a vital interferon regulatory factor in the body reactions of virus infection, could play a pivotal role in the molecular mechanisms of Inflammation, proliferation and differentiation of lymphocytes^13^. By integrating the regulatory relationships and functions among the gene sets of defense response, CEBPB, IRF protein family, and the transcription factor SP1, the etiopathogenesis of leukopenia can be explored more comprehensively.

The third pattern indicated that gene sets affected the side effects and meanwhile are regulated by post-transcription of miRNAs and pre-transcription of TFs. This pattern contained 396 side effect-gene sets regulatory relationships formed by 99 side effects and 53 gene sets. The 53 gene sets were regulated by 198 miRNAs and 315 TFs, which meant each gene set was controlled by 13.65 miRNAs and 29.58 TFs. In Fig. 5C, these TFs (SP1, NFKB1 and RELA) and miRNAs (hsa-miR-7, hsa-miR-125a, hsa-miR-135b) could influence the signal transduction of cell surface receptor and the occurrence of allergic reaction. Besides, the signal pathway of phospholipase C and phosphatidylinositol (PI) 3-kinase has been shown to be highly related with the fatal case in allergic reaction—mast cell activation^14^. Furthermore, SP1 participates in numerous cellular processes, especially, immunoreaction^12^, for example, Leukotriene C(4) synthase (LTC(4)S) is responsible for the biosynthesis of cysteinyl leukotrienes that participate in allergic and asthmatic inflammation, Cell-specific transcription of leukotriene C(4) synthase involves a Kruppel-like transcription factor and Sp1^15^. RELA is the most common member of nuclear transcription factor NF-kB family in mammalian cells, the target genes regulated by it include immune related receptors, cytokines, inflammatory cytokines, adhesion molecules and acute phase proteins, so it plays an important role in regulation of immune cell activation, T, B lymphocyte development and a variety of autoimmune disease occurrence^16^. NFKB1 has described links with allergy or inflammation and may even describe the well-established relationship between viral infections and allergic exacerbations or allergy development^17^. Hsa-miR-7 has been shown to be related to delayed-type hypersensitivity reaction in human skin by influencing T cell activation^18^. The upregulated miR-125a contributed to the upregulation of inflammatory IL-β, IL-6, and TNF-α^19^. Hsa-miR-135b is important in regulating additional pro-inflammatory and anti-viral response genes^20^. So the three miRNAs may have important role in regulating immune responses and inflammation in an allergic environment. Consequently, our method presents a novel insight into the mechanism of allergic reaction by merging the signal transduction of cell surface receptor and the functions of above three transcription factors and three miRNAs.

The last pattern indicated that gene sets affected the side effects, while gene sets were negatively regulated by post-transcription of miRNA controlled by TF. 407 SE-gene set pairs consisted of 100 SEs and 57 gene sets. The 57 gene sets were regulated by 117 miRNAs which was also regulated by 146 TFs. For example, anemia related with the gene sets of extracellular region was regulated by hsa-miR-29a, hsa-miR-29b and hsa-miR-29c, and meanwhile MYC and CEBPA regulated the expression of these miRNAs. FGA, FGB and FGG encoded by the target genes of three miRNAs were spread over the extracellular region and constituted three peptide chains of FBI (fibrinogen) which could disintegrate into fibrin and other split products. It has been widely known that FBIs can cause vasoconstriction, thrombosis, blockage of the blood, and consequently result in local tissue ischemia^12^. Besides, the content of fibrinogen in blood could be changed with the expressions of FGA, FGB and FGG. In fact, miR-29 family members have been investigated the regulating effect on blood disease such as leukemia, which plays a key role in cell growth, proliferation, and apoptosis^21^. And overexpression, rearrangement and translocation of these genes directly regulated by MYC and CEBPA have been associated with a variety of hematopoietic tumors, leukemias and lymphomas^22, 23^. Based on the direction of biological regulation mapping MYC, CEBPA, hsa-miR-29a, hsa-miR-29b, hsa-miR-29c and gene sets of extracellular domain could accurately display the occurrence mechanism of the side effect—anemia.

**Figure 6:**
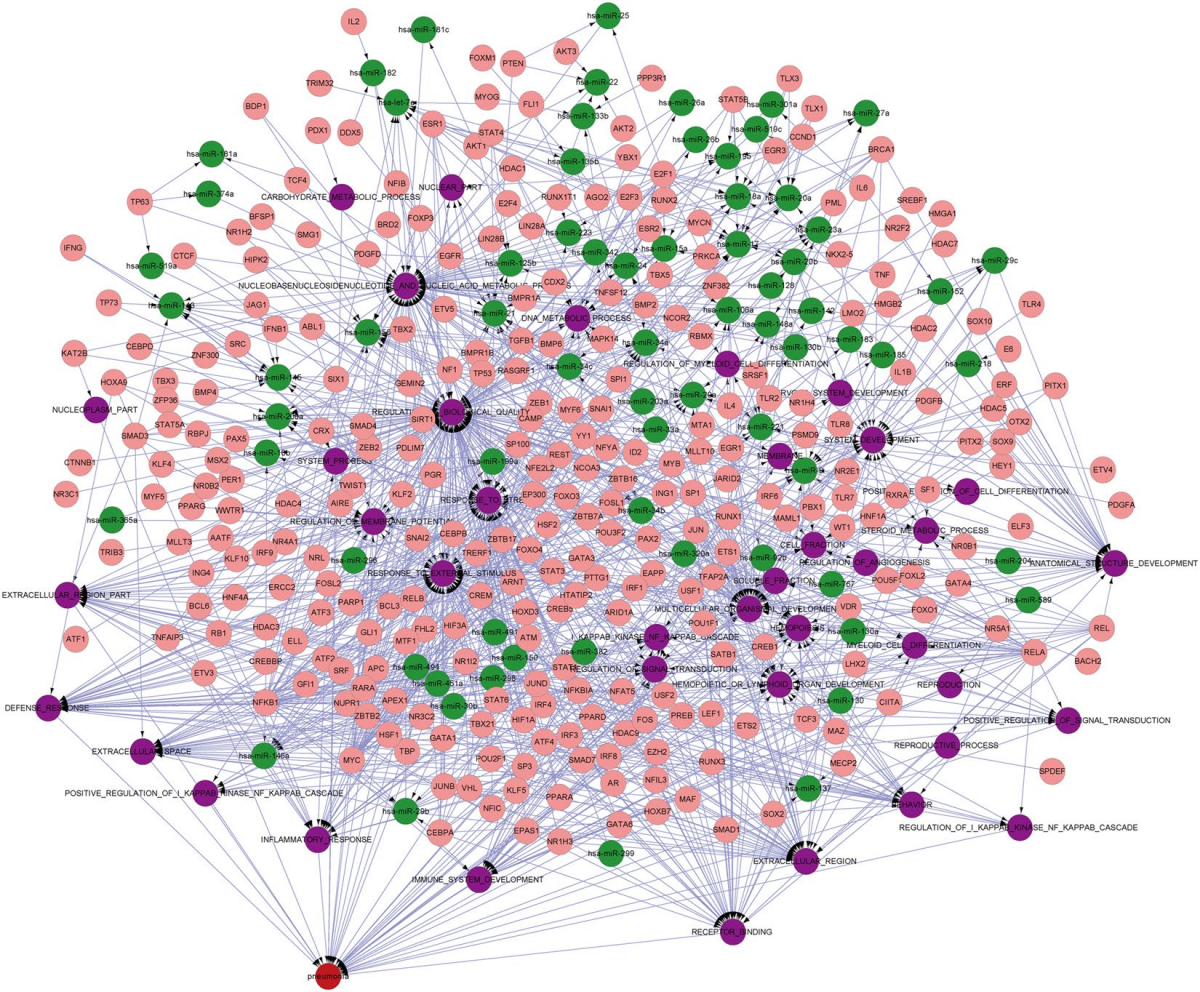
The complex regulatory module of Pneumonia. The red nodes represent side effects; the purple nodes represent gene sets; the green nodes represent miRNAs and the pink nodes represent TFs. Pneumonia, with 39 sets of genes regulated by miRNAs and TF.

**Figure 7:**
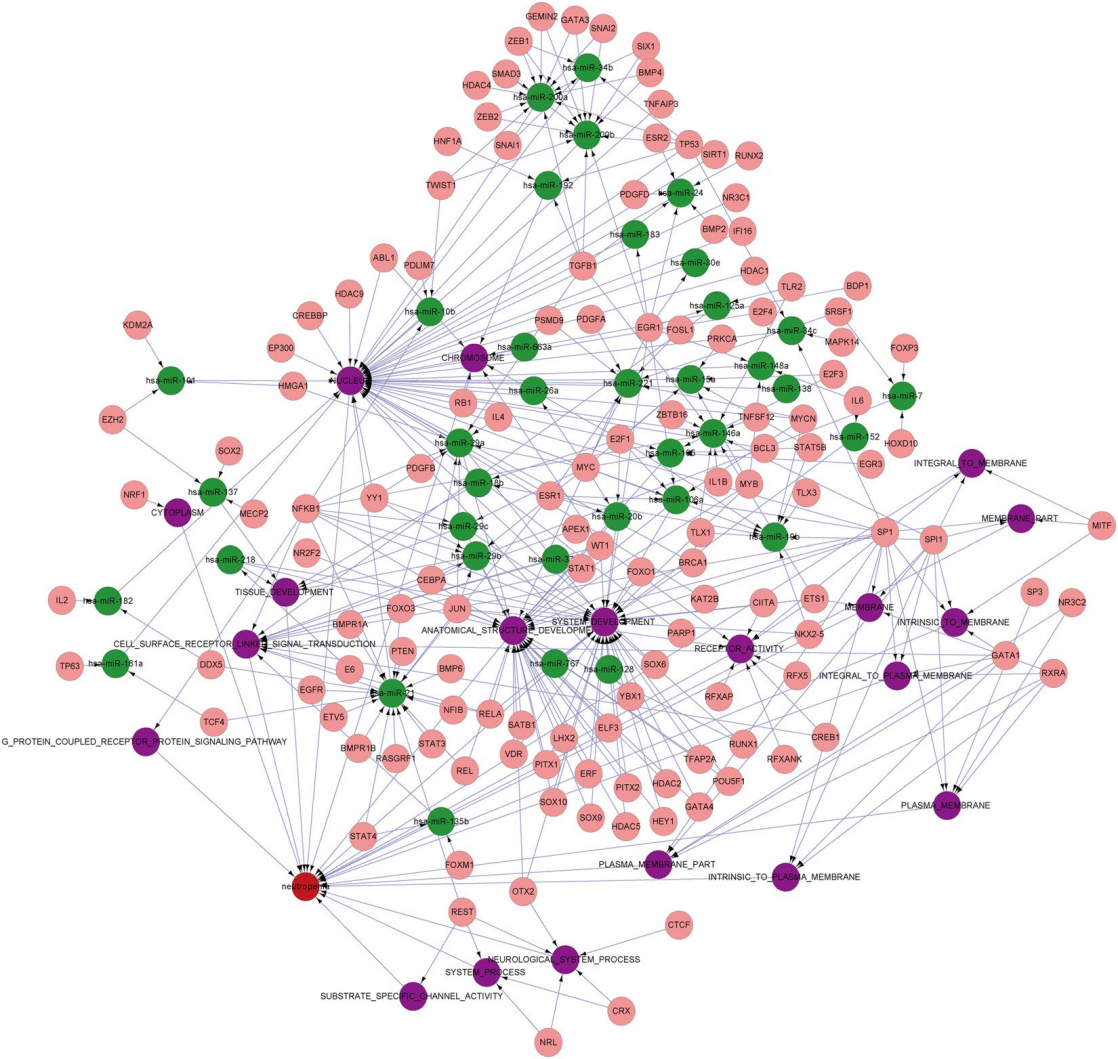
The complex regulatory module of Neutropenia. The red nodes represent side effects, the purple nodes represent gene sets, the green nodes represent miRNAs and the pink nodes represent TFs. Neutropenia, with 20 sets of genes regulated by miRNAs and TFs.

In order to explore the role of complex regulatory network and interpret comprehensively the occurrence mechanism of side effects, Pneumonia (Fig. 6) and Neutropenia (Fig. 7) were extracted from 11 side effects controlled by 4 patterns and took as examples.

Pneumonia is an infection of the lungs that is caused by drugs and bacteria, and affected by receptor mediated endocytosis^24^. It is characterized primarily by inflammation of the alveoli in the lungs or by alveoli that are filled with fluid. The results in this study illustrated that pneumonia was related with gene set of immune system and inflammatory response. Both of the immune system and inflammatory response participated in the process of pneumonia. With the invasion of pathogen, immune system could be activated via the proliferation and differentiation of immune cells. Simultaneously, inflammatory cells gather in the lung tissue and release plenty of inflammatory factors, which induce local inflammatory effects as edema, congestion and cause pneumonia eventually. The results in this study also showed that the gene set of immune system development was regulated by the miRNA hsa-miR-29b, while gene set of inflammatory response was regulated by the miRNA hsa-miR-146a. The human miR-29 is involved in regulation of the innate and adaptive immune responses, and miR-29 family members are up-regulated during influenza A viruses infection which primarily targets respiratory epithelial cells and produces clinical outcomes ranging from mild upper respiratory infection to severe pneumonia^25^. The has-miR-146a could induce inflammatory and anaphylaxis via regulating the immune system and inflammatory response associated with the pneumonia^26^. And the transcription of two miRNAs were regulated by transcription factors NFKB1, CEBPA and MYC etc, which have important role in regulating immune responses and inflammation^22, 23, 27^.

Neutropenia can occur because of decreased production in the bone marrow, increased destruction, marginalization and sequestration, and medications. It is a common adverse event associated with many medicines, especially cytotoxic chemotherapy agents.

The relationship between neutropenia and cell surface receptor linked signal transduction was found in this study. Most cytotoxic chemotherapy exerts its pharmacological activity by acting on cell surface receptor, triggering downstream signal transduction, causing DNA damage in either a cell-specific or cell-nonspecific manner. By damaging the DNA of malignant cells, chemotherapy is able to produce killer malignant cells. Many chemotherapy agents cause bone marrow suppression resulting in neutropenia, which leads to an increased risk of infection^23^. This biological progress could be regulated by hsa-miR-21 which was significantly down-regulated in the anemia and neutropenia^28^. And the interaction between hsa-miR-21 and transcription factor STAT3 regulated by PIAS3 has also been reported^29^, so the hypothesis that the STAT3 could influence the expression of gene set cell surface receptor linked signal transduction via regulation to has-miR-21 is reasonable.

## Discussion

Undoubtedly, the occurrence of any side effect was a complex organism response, and the occurrence mechanism could not be discovered comprehensively if only based on the single gene set, or individual miRNA, or only one regulatory pattern.

In this study, the side effects, miRNAs and TFs through the biology processes were performed integrated analysis, and a framework to explore the mechanism of side effects was provided.

From the side effect-gene set network we found that most of gene sets were only involved in a few side effects, and the gene sets involving in only one side effect which are called the special gene sets are mainly related with lightheadedness, pneumonia, thrombocytopenia and vaginal discharge. In addition, the minority of gene sets was related with multiple side effects, and the gene sets associated with more than 50 side effects were involved in the function of cell membrane. The majority of side effects were regulated by multiple gene sets; more importantly, four side effects related with the maximum gene sets were also regulated by most of special gene sets. Furthermore, the side effects within the same SE-gene set interaction module tended to share gene sets and occur concomitantly.

**Figure 8:**
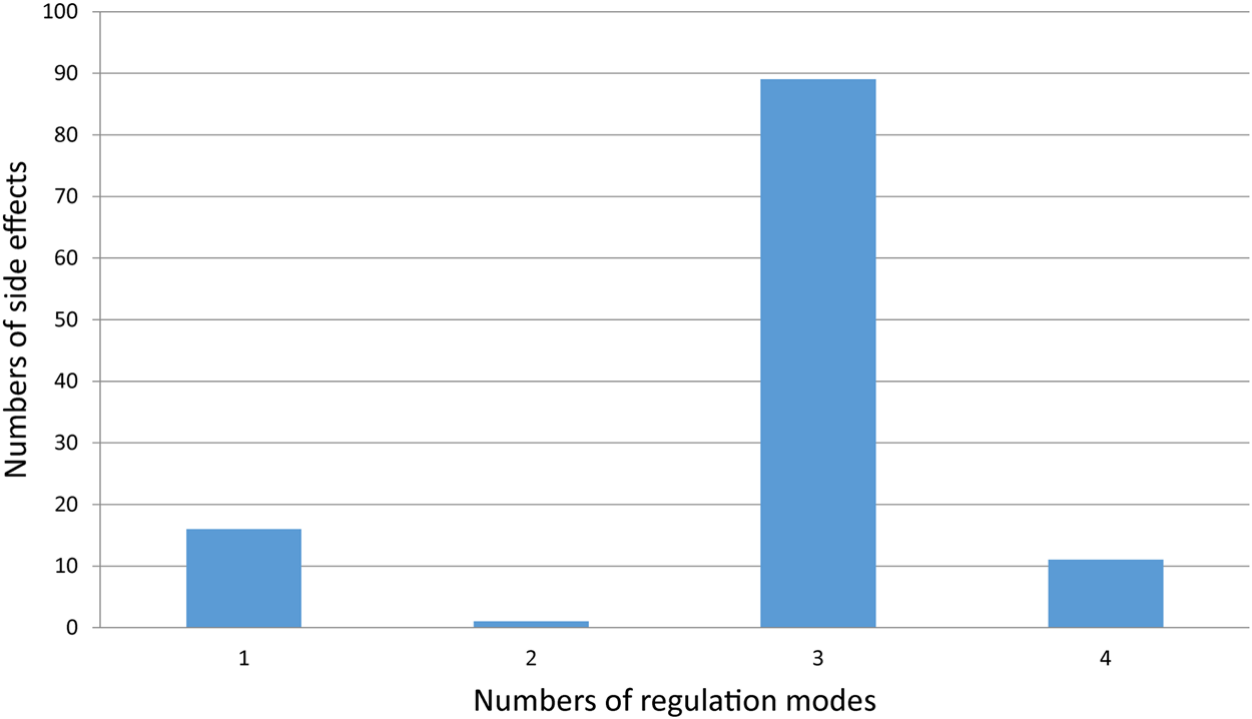
The distribution of side effects under different number of regulation patterns. The horizontal axis represents the number of regulation modes, and the vertical axis shows the number of side effects. For example, there are 16 side effects regulated by 1 regulation mode.

Utilizing the SE-gene set-miRNA (TF) composite regulatory network, we extracted complex regulatory modules including four regulation patterns: SE-gene set-miRNA relationships, SE-gene set-TF relationships,SE-gene set-miRNA (and TF) and SE-gene set-miRNA-TF regulative relationships,and 117 side effect-related module sets. The relations between 117 side effects and the number of regulatory patterns were summarized (Supplementary Data 7), and only 16 SEs was identified to be related with one regulatory pattern accounting for 13.7% of the total side effects. Besides, at least two regulatory patterns were identified to be involved in the occurrence of the remaining 101 SEs which occupied 86.3% of the total side effects. 89 of 101 SEs were related with three patterns and constituted 76.1% to all the side effects. Figure 8 showed that most side effects were the productions of multiple regulatory patterns which were complex controlled by numerous gene sets, miRNAs and TFs. miRNAs and TFs regulated gene sets state through their target proteins respectively and influenced each other at the mean while. Consequently, based on these regulatory modules from the complex regulatory network composed of gene sets, miRNAs and TFs, exploring the occurrence of side effects could benefit researchers to get a deeper understanding about the occurrence mechanism of side effects *in vivo*.

To ensure the authenticity of the data and objectivity of results. All the target data of miRNAs, TFs and TF-miRNA regulatory relationships has been validated by existing experimental studies. However, that induced the incompleteness of data. With the improvement of target data, miRNAs and TFs, there will be more broad application space for our strategy.

## Materials and Methods

### Data extraction

Drug-SE relationship pairs were extracted from side effect resource (SIDER)^30^ which contains the frequency of occurrence of drug-SE pairs. However, drug-side effect relationships need to be filtered to find highly occurring relationships of gene expression data, because side effects do not occur in gene expression data every time^1^. So, in order to ensure the accuracy of the results, twenty percent was set as a threshold of frequency to find drug-side effect relationships based on empirical probability knowledge. The frequency was more than 20% of drug-side effect relationships was selected for further study, and 716 drug-SE relationships including 72 drugs and 169 SEs were obtained eventually.

The genes significantly related with drugs were derived based on the probe-rank information of 6100 gene chips which was related with 1309 drugs, from Connectivity Mapping (CMAP) supplied by Francesco Iorio *et al*.^31^. They obtained a single Prototype Ranked List (PRL) of genes for each drug in the dataset. The PRL captures the consensus transcriptional response of a compound across different experimental settings, consistently reducing irrelevant effects due to toxicity, dosage, and cell line. After the rank order, the first 250 genes at the top of the PRL (most overexpressed) and the last 250 genes at the bottom of the PRL (most downregulated) were considered the “optimal” gene signature for each drug. Here, we utilized their ranked gene expression profiles of drugs, and then selected the top 250 genes of the PRL and the last 250 of the PRL genes as the significant related genes of drugs.

The biological processes were obtained from the Molecular signatures database (MSigDB) 3.0^32^, which provided 1454 GO gene sets including 825 GO biological processes, 233 GO cellular components and 396 GO molecular function. The 6422 experimentally validated human miRNA-target gene pairs were obtained from the miRTarBase^33^, TarBase^34^ and miRecords^35^.

8215 human TF-target gene pairs were extracted from TRRUST^36^, and 651 human TF-miRNA regulatory pairs from the real TF-miRNA regulation database TransmiR were established by Juan Wang^37^.

### Construction of SE-gene sets network

To acquire the significantly associated gene sets of drugs, the differentially expressed genes were selected for each drug based on the row-rank matrix of gene chip, and then the differentially expressed genes were enriched into GO gene sets using the method of fisher enrichment analysis. The p-value was calculated according to the formula as follows, and adjusted by FDR (cutoff: 0.05)^38^.

1 $${\rm{p}}=1-\sum _{i=0}^{x-1}\frac{(\begin{array}{c}M\\ i\end{array})(\begin{array}{c}N-M\\ K-i\end{array})}{(\begin{array}{c}N\\ K\end{array})}\,$$

N: The number of genes in genome-wide gene in total

M: The number of genes in the functional genes

K: The number of differential expression genes of drugs

x: The gene intersections between differential expression genes and functional genes

Using the SE-drugs relationships and drug-gene set relationships, composite relationships of SE-drug-gene set were combined, in which drugs acted as a medium. In order to discover how many drugs sharing the same gene set could indicate the significant association between side effects and gene sets, the SE-drug relationships were randomly re-arranged. Firstly, the side effect related with at least two drugs were selected, and number (n) of drugs associated with these side effects were acquired. Secondly, n drugs from 72 drugs were randomly selected, and the number of common drugs of gene sets related with these n drugs were recorded. This step was repeated for 1000 times. In this step, the number distribution of drugs related with gene sets determined the number of related drugs with gene sets (p = 0.05)^1^. For every composite relationship pair of SE-drug-gene set, the gene set was significantly related with SE when the number of drugs related with the gene set was equal or larger than the threshold under the random perturbation. Afterwards, SE-gene set network was constructed.

### Construction of SE-gene set-miRNA/TF complex network

Since miRNAs and TFs can regulate expression of more than one gene during the real biological processes, thus the gene sets were enriched into miRNA and TF, and the SE-gene set-miRNA/TF network was generated by combining the SE–gene set relationships and gene set-miRNA (TF) relationships. The fisher enrichment analysis were carried out, and the enrichment P-values were adjusted by FDR^38^. Based on the TF-miRNA relationships obtained from TransmiR database, the side effect-gene sets-miRNA network and side effect-gene sets-TF network were mapped, and then a directed complex network including four kinds of regulations were constructed.

Each of side effects, extracted SE-related gene sets, gene set-related miRNAs and TFs in the SE-gene set-miRNA (TF) complex network were analyzed. The direct complex regulatory relationship consisting of gene sets, miRNAs and TFs could act as a complex module to regulate the side effect.

## Electronic supplementary material

Supplementary information

Supplementary Data
