# Supplementary material for: Large-Scale Analysis of Drug Side Effects via Complex Regulatory Modules Composed of microRNAs, Transcription Factors and Gene Sets
Source: Sci Rep. 2017 Jul 20;7:5962. doi: 10.1038/s41598-017-06083-5 (PMC5519677; doi:10.1038/s41598-017-06083-5)
Supplement: Supplementary file 1 — Supplementary information [file 41598_2017_6083_MOESM1_ESM.pdf]

Large-Scale Analysis of Drug Side Effects via Complex Regulatory Modules Composed of microRNAs, Transcription Factors and Gene Sets

Xiaodong Jia[1,2‡], Qing Jin[1‡], Xiangqiong Liu[1, ‡], Xiusen Bian[1‡], Yunfeng Wang[1], Lei Liu[1], Hongzhe Ma[1], Fujian Tan[1], Mingliang Gu[2,3,] *, Xiujie Chen[1,]*

1.College of Bioinformatics Science and Technology, Harbin Medical University, Harbin, China.

2.Joint Laboratory for translational medicine research, Beijing Institute of Genomics, Chinese Academy of Sciences & Liaocheng People's Hospital, Liaocheng, China.

3.CAS Key Laboratory of Genome Sciences and Information, Beijing Institute of Genomics, Chinese Academy of Sciences(CAS), Beijing, China.

‡Joint first authors

***** Corresponding authors

Supplementary Data Lists

Supplementary Data1: Pairs of the drug-gene set network

Supplementary Data2: Pairs of the SE-related gene set network

Supplementary Data3: The single-gene set regulated SE

Supplementary Data4: The single-SE related gene sets

Supplementary Data5: Pairs of the SE-gene set-miRNA (TF) complex network

Supplementary Data6: The regulatory relationship of 117 SEs

Supplementary Data7: SE-related regulatory patterns

Supplementary Fig 1: The SE-related gene set network

Supplementary Fig 2: The SE-gene set-miRNA (TF) complex network

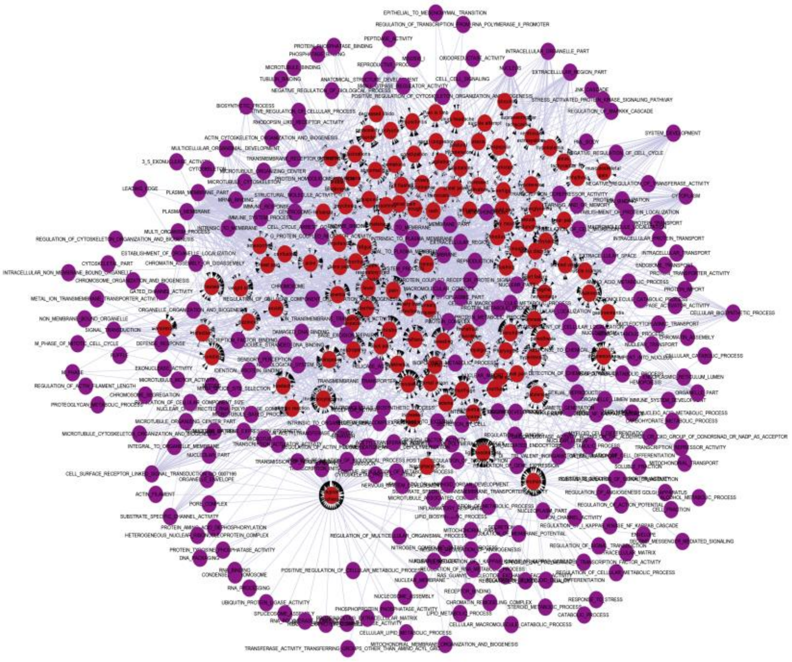

Supplementary Fig 1: The SE-related gene set network

The SE-gene set network included 117 SEs, 256 gene sets and 2064 SE-gene set relationships. The red nodes represent side effects and the purple nodes represent gene sets.

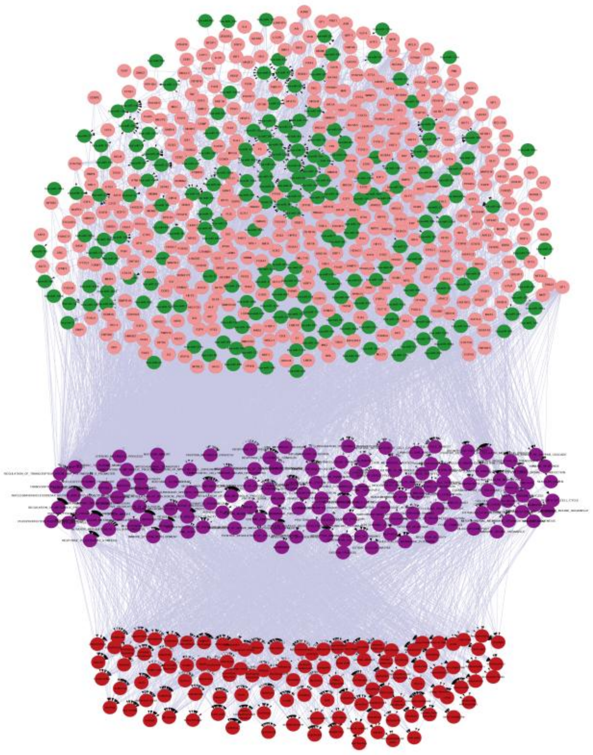

Supplementary Fig 2: The SE-gene set-miRNA (TF) complex network

The SE-gene set-miRNA (TF) complex network included 117 SEs, 142 gene sets, 198

miRNAs, 404 TFs，1671 SE-gene set pairs, 1072 gene set-miRNA pairs， 2480 gene set-TF pairs and 466 TF-miRNA regulatory pairs. For each side effect, SE -special complex regulatory module was extracted from the SE-gene set-miRNA (TF) complex network. The red nodes represent side effects; the purple nodes represent gene sets; the green nodes represent miRNAs and the pink nodes represent TFs.
